# Long-term antibody persistence against hepatitis B in adolescents 14–15-years of age vaccinated with 4 doses of hexavalent DTPa-HBV-IPV/Hib vaccine in infancy

**DOI:** 10.1080/21645515.2018.1509658

**Published:** 2018-09-11

**Authors:** Tino F. Schwarz, Ulrich Behre, Thomas Adelt, Matthias Donner, Pemmaraju V. Suryakiran, Winnie Janssens, Narcisa Mesaros, Falko Panzer

**Affiliations:** a Institute of Laboratory Medicine and Vaccination Centre, Klinikum Würzburg Mitte, Standort Juliusspital, Würzburg, Germany; b Pediatric Practice, Kehl, Baden-Württemberg, Germany; c Pediatric Practice, Bramsche, Germany; d Pediatric Practice, Mönchengladbach, Germany; e GSK, Bangalore, India; f GSK, Wavre, Belgium; g Pediatric Practice, Mannheim, Germany

**Keywords:** hepatitis B, anamnestic response, challenge dose, DTPa-HBV-IPV/Hib, immune memory, long-term persistence, seroprotection, adolescents

## Abstract

We evaluated antibody persistence against hepatitis B virus (HBV) in adolescents previously vaccinated with a hexavalent diphtheria-tetanus-acellular pertussis-HBV-inactivated poliovirus-*Haemophilus influenzae* type b conjugate vaccine (DTPa-HBV-IPV/Hib), as part of the national newborn immunization program in Germany. We also assessed the anamnestic response to a challenge dose of a monovalent HBV vaccine. In this phase 4, open-label, non-randomized study (NCT02798952), 302 adolescents aged 14–15 years, primed in their first 2 years of life with 4 DTPa-HBV-IPV/Hib doses, received one challenge dose of monovalent HBV vaccine. Blood samples were taken before and one month post-vaccination and used to determine antibody levels against hepatitis B surface antigen (HBs). Reactogenicity and safety were also assessed post-challenge dose. Pre-challenge dose, 53.7% of 268 participants included in the according-to-protocol cohort for immunogenicity had anti-HBs antibody concentrations ≥10 mIU/mL (seroprotection cut-off) and 16.8% had anti-HBs antibody concentrations ≥100 mIU/mL. One month post-challenge dose, 93.3% of adolescents had anti-HBs antibody concentrations ≥10 mIU/mL and 87.3% had antibody concentrations ≥100 mIU/mL. An anamnestic response was mounted in 92.5% of adolescents. Injection site pain (in 33.6% of participants) and fatigue (30.2%) were the most frequently reported solicited local and general symptoms, respectively. Six of the 55 unsolicited adverse events reported were considered vaccination-related. Two vaccination-unrelated serious adverse events were reported during the study. Long-term antibody persistence against hepatitis B was observed in 14–15 years old adolescents previously primed in infancy with DTPa-HBV-IPV/Hib. A challenge dose of monovalent HBV vaccine induced strong anamnestic response, with no safety concerns.

## Introduction

Hepatitis B is a potentially life-threatening disease caused by the hepatitis B virus (HBV) with a high burden of disease; approximately 257 million people worldwide are estimated to be infected with HBV.^1^ Although the prevalence of HBV is relatively low in Europe compared with other regions, around 60,000 deaths per year are due to hepatitis B-related liver cancer and cirrhosis.^2^ Children under 5 years of age infected with HBV are at high risk of developing a chronic infection later in life.^3^ In 2015, 63.5% of the 24,573 HBV infection cases reported from 30 European states were chronic infections.^4^

Vaccination against HBV starting at birth is the main preventive measure recommended by the World Health Organization.^5^ In Europe, most countries have included hepatitis B vaccination in their routine childhood immunization program as a 3- or 4-dose schedule, to be completed in the first 2 years of life.^6–8^ Vaccination within the first 12–24 hours from birth is also implemented in Europe, although in most countries a dose at birth is only recommended for ‘at-risk’ populations, such as infants born to HBV-infected mothers or with unknown immune status.^6^

Several monovalent or combination vaccines that prevent HBV infection are currently marketed in Europe. In Germany, the monovalent recombinant hepatitis B vaccine (*Engerix-B Kinder*, GSK) was introduced in the national immunization program in 1995 as a 3-dose vaccination schedule administered at 0, 1, and 6 months of age. Long-term persistence studies showed that immunity to hepatitis B in adolescents vaccinated according to this program persists up to 15–16 years of age.^9,10^ In 2000, the monovalent vaccine was replaced with combination hexavalent vaccines in the German national immunization program. Since 2000, the hexavalent diphtheria-tetanus-pertussis-HBV-inactivated poliomyelitis and *Haemophilus influenzae* type b conjugate combination vaccine (DTPa-HBV-IPV/Hib, *Infanrix hexa*, GSK) is approved for use in Germany. The vaccine is administered as a 3 + 1 vaccination schedule, with the primary doses given at 2, 3, and 4 months of age, followed by a booster dose at 11–14 months of age. Catch-up vaccination is only considered for children and adolescents 2–17 years of age with a missing or incomplete vaccination series in infancy.^11^

The HBV component of DTPa-HBV-IPV/Hib vaccine induces seroprotective levels and antibody concentrations against hepatitis B surface antigen (HBs) comparable to those achieved in individuals who had received the monovalent HBV vaccine.^12^ Studies conducted after primary immunization with either the monovalent or combination hepatitis B vaccine have shown a long-term antibody persistence and immune memory against hepatitis B,^13^ extending up to 20 years after primary vaccination completion.^14^

This is the last of a series of 4 studies aimed to assess the persistence of hepatitis B antibodies from childhood to 14–15 years of age conferred by 4 doses of DTPa-HBV-IPV/Hib administered in the first 2 years of life. Results at 4–5 years,^15^ 7–8 years,^16^ and 12–13 years^17^ of age were previously reported. Each study enrolled different individuals, as they aimed to provide a challenge dose and evaluate the anamnestic response. In the present study, the ability of 14–15-year-olds to mount an anamnestic response to a challenge dose of the monovalent pediatric vaccine, together with safety and reactogenicity were evaluated.

**Figure 1.**
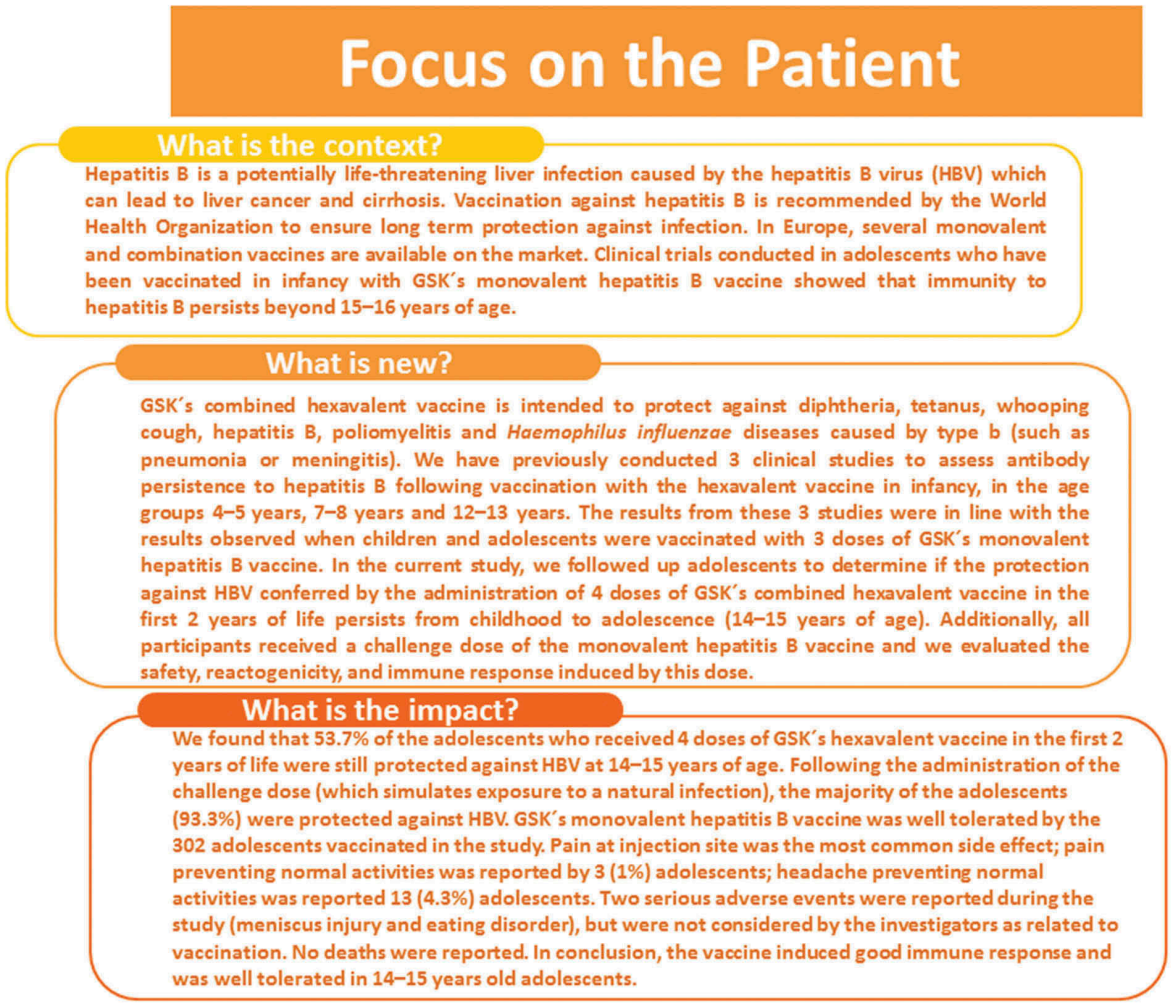
Focus on Patient Section.

A lay language graphical summary contextualizing the results, the potential clinical research relevance and the impact of our study is displayed in the *Focus on Patient* Section (Figure 1).

## Results

### Demographics

**Figure 2.**
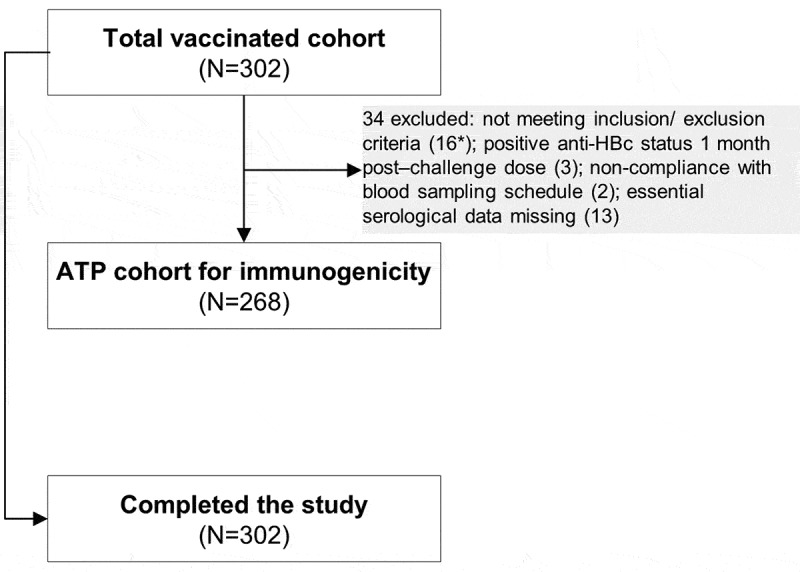
Participant flow chart. ATP, according-to-protocol; N, number of participants; HBc, hepatitis B core antigen.Note: *One participant also had positive anti-HBc status 1 month post-challenge dose.

In total, 302 adolescents previously primed with 4 doses of DTPa-HBV-IPV/Hib during infancy were vaccinated with the monovalent hepatitis B vaccine in the present study and 268 adolescents were included in the according-to-protocol (ATP) cohort for immunogenicity (Figure 2). The mean age at the receipt of the challenge dose was 14.4 years and the majority of the participants were of European heritage (Table 1).

**Table 1.** Characteristics of study participants.

	TVC (N = 302)	ATP cohort for immunogenicity (N = 268)
Mean age ± SD, years	14.4 ± 0.5	14.4 ± 0.5
Male, n (%)	160 (53.0)	137 (51.1)
Geographic ancestry, n (%)		
African heritage/African American	2 (0.7)	2 (0.7)
Asian – Central/South Asian Heritage	1 (0.3)	1 (0.4)
Asian – South East Asian Heritage	1 (0.3)	1 (0.4)
White – Arabic/North African Heritage	4 (1.3)	4 (1.5)
White – Caucasian/European Heritage	293 (97.0)	259 (96.6)
Caucasian/North African Mixture	1 (0.3)	1 (0.4)
Mean weight ± SD, kg	63.2 ± 15.4	62.9 ± 15.1
Mean height ± SD, cm	168.4 ± 9.0	168.1 ± 9.0
Mean body mass index ± SD, kg/m^2^	22.2 ± 4.7	22.2 ± 4.6

TVC, total vaccinated cohort; N, number of participants in each cohort; ATP, according-to-protocol; SD, standard deviation; n (%), number (percentage) of participants in each category.

### Antibody persistence

In the ATP cohort for immunogenicity, before the challenge dose 53.7% of the participants had anti-HBs antibody concentrations ≥10 milli-International Units (mIU)/mL (the accepted correlate of protection), and 16.8% had anti-HBs antibody concentrations ≥100 mIU/mL, which provides a more conservative estimate of protection against hepatitis B infection following vaccination. The antibody geometric mean concentration (GMC) was 15.6 mIU/mL (95% confidence interval [CI]: 12.8–19.1) (Table 2).

**Table 2.** Seropositivity/seroprotection rates and antibody GMCs, overall and by pre-challenge status (ATP cohort for immunogenicity).

		Seropositivity/seroprotection rate (% [95% CI])			
	N	≥6.2 mIU/mL	≥10 mIU/mL	≥100 mIU/mL	GMC (95% CI)
Pre-challenge	268	60.8 (54.7–66.7)	53.7 (47.6–59.8)	16.8 (12.5–21.8)	15.6 (12.8–19.1)
One month post-challenge	268	95.1 (91.8–97.4)	93.3 (89.6–96.0)	87.3 (82.7–91.1)	1975.7 (1436.1–2718.1)
By pre-challenge status					
<6.2 mIU/mL	105	87.6 (79.8–93.2)	82.9 (74.3–89.5)	68.6 (58.8–77.3)	224.7 (143.0–353.1)
≥6.2 – <10 mIU/mL	19	100 (82.4–100)	100 (82.4–100)	100 (82.4–100)	1661.0 (1092.0–2526.4)
≥10 mIU/mL	144	100 (97.5–100)	100 (97.5–100)	99.3 (96.2–100)	9865.7 (7418.8–13,119.5)

ATP, according-to-protocol; GMC, geometric mean concentration; N, number of participants with available results; CI, confidence interval; IU, international units.

### Post-challenge immunogenicity

One month after the challenge dose with the monovalent hepatitis B vaccine, in the ATP cohort for immunogenicity, the percentage of seroprotected participants was 93.3%; 87.3% of adolescents had anti-HBs antibody concentrations ≥100 mIU/mL. Overall, the anti-HBs antibody GMCs increased 126.6-fold from the pre-vaccination level. A higher GMC was observed in adolescents who were seroprotected before vaccination compared to those who had anti-HBs antibody concentrations <10 mIU/mL prior to challenge vaccination (Table 2).

An overall anamnestic response to the challenge dose was observed in 92.5% of adolescents. When the response was stratified based on pre-vaccination antibody levels, 82.9% of initially seronegative participants (anti-HBs antibody concentrations below the assay cut-off of 6.2 mIU/mL) achieved post-vaccination concentrations ≥10 mIU/mL. For the initially seropositive participants, 100% of those with pre-vaccination levels between 6.2–10 mIU/mL and 98.6% (all but 2) of participants with pre-vaccination levels ≥10 mIU/mL mounted an anamnestic response (Table 3).

**Table 3.** Number and percentage of participants with anamnestic response to the challenge dose, overall and by pre-challenge status (ATP cohort for immunogenicity).

	N	n	% (95% CI)
Overall	268	248	92.5 (88.7–95.4)
By pre-challenge status (anti-HBs antibody concentration)			
Seronegative (<6.2 mIU/mL)	105	87	82.9 (74.3–89.5)
Seropositive (≥6.2 mIU/mL)	163	161	98.8 (95.6–99.9)
≥6.2–<10 mIU/mL	19	19	100 (82.4–100)
≥10 mIU/mL	144	142	98.6 (95.1–99.8)

ATP, according-to-protocol; N, number of participants with available results; n (%), number (percentage) of participants with anamnestic response; CI, confidence interval; HBs, hepatitis B surface antigen

Note: Anamnestic response was defined as anti-HBs concentrations ≥10 mIU/mL in participants seronegative (anti-HBs antibody concentrations <6.2 mIU/mL) before the challenge dose and as a ≥ 4-fold increase in anti-HBs concentrations in participants seropositive (anti-HBs antibody concentrations ≥6.2 mIU/mL) before the challenge dose.

### Reactogenicity and safety of the challenge dose

**Figure 3.**
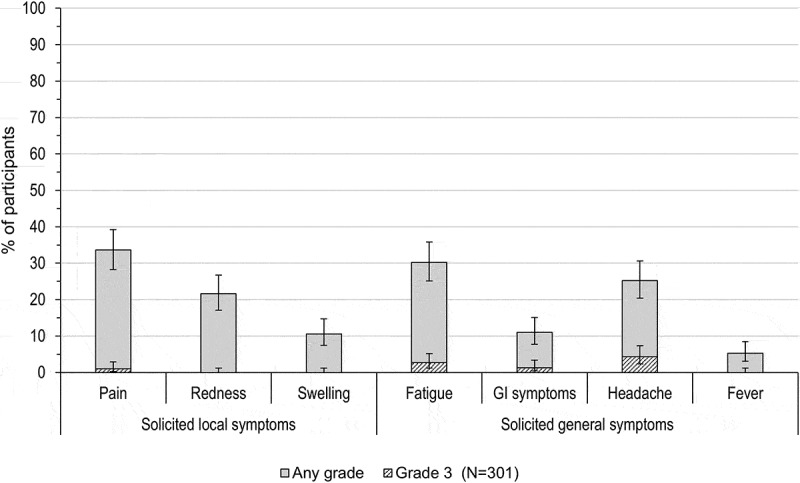
Percentage of adolescents with solicited local and general symptoms (total vaccinated cohort). GI, gastrointestinal; Fever, temperature ≥ 37.5°C; N, number of participants with documented dose.Note: Grade 3 events were defined as preventing normal daily activities (for pain, fatigue, GI symptoms and headache), surface >50 mm (for redness, swelling) and temperature >39°C (fever). Error bars represent 95% confidence intervals.

During the 4-day follow-up period post-challenge dose, at least one unsolicited adverse event (AE)/solicited symptom (local or general) was reported in 65.6% of the vaccinated adolescents. The most frequently reported solicited symptoms were injection site pain (33.6% of participants) and fatigue (30.2%) (Figure 3). Grade 3 injection site pain was reported in 3 (1.0%) adolescents. Solicited general symptoms of grade 3 intensity were reported in ≤ 13 (4.3%) participants; headache was the most frequently reported grade 3 solicited general symptom (Figure 3).

Medical advice was sought by 1 (0.3%) participant with a solicited general symptom and 3.0–16.6% of general symptoms were assessed by the investigator as related to vaccination. Fatigue was the most frequently reported vaccination-related general symptom.

During the 31-day period post-challenge dose, at least one unsolicited AE was reported for 55 (18.2%) of the vaccinated adolescents. The most frequently reported unsolicited AE was upper respiratory tract infection, documented in 10 (3.3%) participants. At least one grade 3 unsolicited AE was reported for 7 (2.3%) adolescents. Unsolicited AEs considered by the investigator to be causally related to vaccination were recorded in 6 (2.0%) participants; these were dizziness (reported for 2 [0.7%] participants) and injection site pruritus, malaise, pain and pain in extremity (each reported for 1 [0.3%] participant).

Two serious AEs (SAEs), meniscus injury and eating disorder, were reported 13 days and 10 days post-challenge dose and neither of them was considered vaccination-related by the investigator. Both were recovered/resolved by study end. No fatal SAEs were reported and no AEs led to withdrawal from the study.

## Discussion

This study was conducted as part of a long-term surveillance program of vaccination with DTPa-HBV-IPV/Hib, to assess persistence of immune response to vaccination against HBV from infancy to adolescence, in individuals who had received 4 doses of DTPa-HBV-IPV/Hib during the first 2 years of life in German clinical practices.

We observed that following vaccination with DTPa-HBV-IPV/Hib in infancy, anti-HBs antibodies persisted up to the age of 14–15 years, with 53.7% of participants maintaining seroprotective antibody levels in their adolescence. In the previous 3 studies in children and adolescents primed with DTPa-HBV-IPV/Hib in the first 2 years of life, anti-HBs antibodies persisted in 85.3% of children aged 4–5 years,^15^ 72.2% of children 7–8 years of age,^16^ and in 60.5% of adolescents aged 12–13 years,^17^ showing an expected decrease in seroprotective antibody levels with increasing age. Nevertheless, as shown by our results, more than half of the participants can still retain seroprotective levels against HBV at 14–15 years of age. Although 46.3% of participants in our study did not maintain antibody concentrations ≥10 mIU/mL in adolescence, there is ample evidence showing that even in the absence of seroprotective antibody levels, protection against hepatitis B disease upon exposure to the virus is mounted by an anamnestic response.^18,19^ Our results compare well with previous studies in which antibody persistence was evaluated in German adolescents who had received 3 doses of the monovalent pediatric hepatitis B vaccine during infancy, which showed that 65.4% of the adolescents maintained seroprotective levels against HBV at 15–16 years of age.^10^ This suggests that the use of the hexavalent combination vaccine does not impact the immune response against hepatitis B over long periods of time.

We observed a robust immune response in participants challenged with a dose of monovalent hepatitis B vaccine (mimicking natural exposure to the virus), with 87.3% of adolescents reaching anti-HBs antibody concentrations ≥100 mIU/mL. Although the seroprotection percentage was slightly lower than values observed when the challenge dose was administered at 7–8 years of age (98.9% of participants having anti-HBs antibody concentrations ≥10 mIU/mL)^16^ or 12–13 years of age (97.6% participants had anti-HBs antibody concentrations ≥10 mIU/mL),^17^ the vast majority of adolescents in our study (93.3%) were protected against HBV following the administration of a challenge dose at 14–15 years of age. A waning of immunity and, in particular, of immune memory with increasing time from primary vaccination against HBV during the first years of life has been described before^20-23^ and might reflect a decrease in antigen-specific memory B-cells over time. However, an anamnestic response to the hepatitis B challenge dose, which is most likely to provide protection against chronic disease in view of the high immune tolerance period for chronic HBV infections^24^ was mounted by 92.5% of participants. In the previous studies, an anamnestic response to the challenge dose was observed in ≥ 96.5% of participants at younger ages,^15–17^ suggesting that increasing age does not impact significantly the immunologic memory to vaccination. Moreover, a study in German children and adolescents vaccinated with various vaccines against HBV during infancy showed that, although seroprotective anti-HBs antibody levels were sustained in only half of them at 6–14 years following vaccination, around 90% of participants mounted an immune response to a booster dose of monovalent HBV vaccine.^21^ The comparison to our results is limited by the difference in study setting, age groups, previous immunization and timeframe from vaccination during infancy; however, both studies suggest that boostability of immune response against HBV can be achieved during adolescence.

When the results were evaluated based on the pre-challenge status, approximately 7% of participants in our study who were seronegative prior to vaccination did not achieve seroprotective antibody concentrations following the challenge dose and can be considered non-responders. A small number of non-responders was also observed at 12–13 years of age.^17^ Remarkably, among adolescents who were seropositive at pre-challenge dose, all but 2 mounted a seroprotective antibody response.

The reactogenicity and safety profile of the monovalent hepatitis B vaccine challenge dose were consistent with the well-known tolerability and safety profile of the vaccine in all age categories^25,26^ and in the previous 3 related studies.^15–17^

The potential limitations of the study included its open label, non-randomized design. The trial was conducted in only one country, although the results can be easily generalized to European populations with similar HBV prevalence and immunization practices. As the production of antigen-specific memory B-cells might not necessarily diminish with the decrease in anti-HBs antibody levels over time,^19^ the evaluation of cell-mediated immune response would have been of interest; therefore, this is another limitation of our study. In addition, the lack of data on post-primary and post-booster immune response in infancy hindered the estimation of the true decline in antibody levels. This also prevented us from identifying non-responders to vaccination during the first 2 years of life, which could have further clarified the response or lack thereof to HBV vaccination during adolescence. Antibodies against hepatitis B core antigen (anti-HBc) were only measured post-challenge dose, therefore we could not conclude when the infection with HBV occurred for the 4 participants with positive anti-HBc status. Determining the timing of the HBV infection would have been of value, especially if it occurred after the full course of primary immunization, and so the lack of anti-HBc data at multiple timepoints is another limitation of the study.

In conclusion, long-term antibody persistence against HBV was observed in 14–15 years-old adolescents who received 4 doses of DTPa-HBV-IPV/Hib during infancy and a strong anamnestic response to a challenge dose of monovalent hepatitis B vaccine was mounted in the majority of participants, with no safety concerns identified.

## Methods

### Study design and participants

This phase 4, open-label, non-randomized study was conducted in 14 centers in Germany, between August 2016 and July 2017. Participants were healthy children aged 14–15 years, vaccinated with 4 doses of DTPa-HBV-IPV/Hib in their first 2 years of life as part of the national immunization program in Germany (*i.e*., 3 primary doses before 9 months of age and a booster dose between 11 and 18 months of age), from whom both informed assent and informed consent signed by their parents/legal guardians were obtained. Adolescents with history of hepatitis B disease or those who had received hepatitis B vaccination at birth or a booster vaccination since the administration of the fourth DTPa-HBV-IPV/Hib dose in the second year of life were not eligible for enrolment. Female participants of childbearing potential were only enrolled if they had a negative pregnancy test on the day of vaccination, had practiced adequate contraception for 30 days prior to vaccination and agreed to continue to do so for 2 months after the challenge dose.

All participants received one challenge dose (0.5 mL) of monovalent hepatitis B vaccine (containing 10 µg of HBs) intramuscularly in the deltoid region of the non-dominant arm.

The study was conducted in accordance with the ICH Guideline for Good Clinical Practice, Declaration of Helsinki and all applicable regulatory requirements, and it was registered at www.clinicaltrials.gov (NCT02798952). A protocol summary is available at www.gsk-clinicalstudyregister.com (study ID: 106794).

## Study objectives

The primary objective assessed the percentage of participants with anti-HBs antibody concentrations ≥100 mIU/mL, at 1 month after the challenge dose. Secondary objectives assessed persistence of the immune response to hepatitis B vaccination, in terms of seroprotection status and antibody concentrations at 14–15 years of age, after the receipt of 4 DTPa-HBV-IPV/Hib doses in the first 2 years of life. The immune response elicited by a challenge dose of monovalent hepatitis B vaccine, in terms of anamnestic response, seroprotection status, and antibody concentrations 1 month after vaccination and the vaccine´s safety and reactogenicity were also evaluated.

## Immunogenicity assessments

Blood samples (2.5 mL) were collected before and 1 month post-administration of the challenge dose at 14–15 years of age. Immune responses to hepatitis B were measured using a chemiluminescence immunoassay. Participants with anti-HBs antibody concentrations ≥6.2 mIU/mL (the assay cut-off) were considered seropositive, while anti-HBs antibody concentrations ≥10 mIU/mL indicated seroprotection.

Anamnestic response to the challenge dose was evaluated 1 month post-vaccination and defined as a ≥ 4-fold increase in post-vaccination anti-HBs antibody concentrations for initially seropositive participants or anti-HBs antibody levels ≥10 mIU/mL post-vaccination for initially seronegative participants.

## Safety assessments

Participants were observed for at least 30 minutes following the administration of the vaccine for any immediate reactions. Solicited local and general symptoms occurring within a 4-day (Days 0–3) and unsolicited AEs occurring within a 31-day (Days 0–30) period after vaccination were recorded by the participants parents/legally acceptable representatives on diary cards, which were returned at next visit. All solicited local symptoms were considered as related to vaccination, while the causality of the general solicited symptoms and AEs was assessed by the investigator.

The intensity of all AEs was evaluated on a 3-grade scale from mild to severe. Severe (grade 3) solicited symptoms were defined as diameter >50 mm (for redness, swelling), axillary temperature >39.0°C (fever) or as preventing normal daily activities (for all other solicited symptoms). Related and medically-attended AEs were also recorded. SAEs were recorded up to study end.

## Statistical analyses

The objectives of the study were descriptive. However, for a sample size of 270 evaluable adolescents, the power to obtain a lower limit of the 95% CI greater than 5% below the expected estimate for the percentage of children with anti-HBs antibody concentrations ≥100 mIU/m was ≥ 86%, assuming a true response rate of 95% (based on previous results obtained in children).^15^ Immunogenicity analyses were performed on the ATP cohort for immunogenicity, which included all vaccinated participants from the total vaccinated cohort, who met the eligibility criteria, complied with protocol-defined procedures and for whom immunogenicity results were available post-vaccination. Anti-HBs antibody GMCs and the percentages of children with anti-HBs antibody concentrations ≥6.2 mIU/mL, ≥10 mIU/mL, and ≥100 mIU/mL were calculated with 95% CIs prior and 1 month after vaccination with the challenge dose. The GMC calculations were performed by taking the anti-log of the mean of the log_10_ concentration transformations. Participants with antibody concentrations below the assay cut-off (6.2 mIU/mL) were given an arbitrary value of half the cut-off and those with antibody concentrations between the assay cut-off and the lower limit of quantification (7.65 mIU/mL) were given the value of the assay cut-off. Missing or non-evaluable measurements were not replaced. The percentage of participants (with 95% CI) with anamnestic response to the challenge dose was calculated overall and based on pre-vaccination status.

Safety analyses were performed on the total vaccinated cohort. Missing or non-evaluable participants were not included in the analysis of solicited symptoms and were considered as without an AE in the analysis of unsolicited AEs. The percentage of children with at least one AE, solicited (local and general) symptoms and unsolicited AEs, and SAEs were calculated with 95% CIs.

*Infanrix hexa* and *Engerix-B Kinder* are trademarks of the GSK group of companies.

## Abbreviations

AE: adverse event
ATP: according-to-protocol
CI: confidence interval
DTPa-HBV-IPV/Hib: combined hexavalent diphtheria-tetanus-pertussis- hepatitis B virus -inactivated poliomyelitis and *Haemophilus influenzae* type b conjugate vaccine
GMC: geometric mean concentration
IU: international units
HBs: hepatitis B surface antigen
HBV: hepatitis B virus
SAE: serious adverse event.

## Trial registration

NCT02798952 (www.clinicaltrials.gov)
